# Neurodevelopmental Disorders and Connective Tissue-Related Symptoms: An Exploratory Case-Control Study in Children

**DOI:** 10.3390/children12010033

**Published:** 2024-12-28

**Authors:** Leonardo Zoccante, Gianfranco Di Gennaro, Erika Rigotti, Marco Luigi Ciceri, Andrea Sbarbati, Marco Zaffanello

**Affiliations:** 1 Azienda Ulss 9 Scaligera, 37122 Verona, Italy; leonardo.zoccante@aulss9.veneto.it; 2 Science of Health Department, School of Medicine, Magna Graecia University of Catanzaro, 88100 Catanzaro, Italy; gianfranco.digennaro@unicz.it; 3 Department of Surgery, Dentistry, Paediatrics and Gynaecology, University of Verona, 37126 Verona, Italy; erika.rigotti@aovr.veneto.it; 4 Childhood, Adolescence, Families and Family Health Centers, Via Salvo d’Acquisto 7, 37122 Verona, Italy; marco.ciceri@aulss9.veneto.it; 5 Department of Neurosciences, School of Medicine, Biomedicine and Movement Sciences, Anatomy and Histology Section, University of Verona, 37124 Verona, Italy; andrea.sbarbati@univr.it

**Keywords:** autism spectrum disorder, attention deficit hyperactivity disorder, connective tissue, neurodevelopmental disorders, Tourette syndrome

## Abstract

**Background/Objectives**: Autism spectrum disorder (ASD), attention deficit hyperactivity disorder (ADHD), and Tourette syndrome (TS) are neurodevelopmental disorders (NDDs) with overlapping symptoms, suggesting a partially shared genetic origin. This study investigates the prevalence of connective tissue-related conditions in individuals with ASD, ADHD, or TS. **Methods**: A questionnaire was administered to families of 120 individuals with ASD, ADHD, or TS, collecting sociodemographic data and examining 10 types of disorders affecting various organs and systems. Statistical analyses were performed using STATA 16.0, with the significance level set at 5%. **Results**: Among the 120 patients, 48 had ASD, 36 had ADHD, and 36 had TS. Flat feet were significantly more common in individuals with ASD (52.1%; OR 7.20; *p* < 0.001), ADHD (52.8%; OR 6.73; *p* = 0.001), and TS (38.9%; OR 3.70; *p* = 0.034) compared to controls (13.6%). Hypersensitivity was more frequent in individuals with ASD (56.3%; OR 5.90; *p* = 0.001), ADHD (50.0%; OR 4.11; *p* = 0.011), and TS (58.3%; OR 5.35; *p* = 0.003) compared to controls (18.2%). Myopia and ptosis were more common in ADHD (30.6%). There was a possible trend towards orthodontic device use in TS (OR 3.20; *p* = 0.076). Flat feet and hypersensitivity were also common in fathers (31.0% and 36.4%, respectively), mothers (31.0% and 15.2%), and patients (43.8% and 55%). **Conclusions**: The findings of this study highlight the significant associations between ASD, ADHD, and TS and specific physical symptoms, such as flat feet, sensory hypersensitivity, and other connective tissue-related manifestations. The familial prevalence of these symptoms suggests a potential genetic underpinning, further supporting the hypothesis of shared aetiological pathways. These insights underscore the need for interdisciplinary research to explore the mechanisms linking neurodevelopmental and connective tissue disorders, aiming to improve diagnosis and management strategies.

## 1. Introduction

Autistic spectrum disorder (ASD), attention deficit hyperactivity disorder (ADHD), and Tourette syndrome (TS) are neurodevelopmental disorders (NDDs) that typically emerge during childhood and persist throughout life. ASD is a complex developmental condition that affects how individuals interact, communicate, and behave. ADHD is an NDD characterised by persistent patterns of inattention, hyperactivity, and impulsivity. TS is a tic disorder involving involuntary, repetitive movements (motor tics) and sounds (vocal tics).

These conditions manifest in diverse ways, leading to impairments in personal, social, academic, and occupational functioning [1,2]. The aetiopathogenesis of these disorders is thought to involve a complex interplay of genetic, epigenetic, and environmental factors. Extensive research into the heritability of NDDs suggests that a substantial proportion of phenotypic variance can be attributed to genetic influences [3,4]. Notably, certain genes associated with ASD have also been implicated in ADHD and TS [4].

Although ASD, ADHD, and TS are traditionally viewed as distinct conditions, differences in the timing of abnormal neurodevelopment and, more importantly, the neural circuits involved distinguish these disorders [5]. However, emerging evidence indicates they may share partially overlapping genetic factors [4,6]. Specific genes and genetic regions have been identified that appear to contribute to a shared genetic predisposition for ASD, ADHD, and TS [4]. This genetic overlap is further supported by the significant symptomatological similarities among these disorders, which include attentional difficulties, impulsivity, repetitive or ritualistic behaviours, deficits in communication and social interaction, obsessive traits, anxiety disorders, and abnormalities in sensory processing [1,4,7].

Moreover, ASD, ADHD, and TS seem to exhibit overlapping neuropathological mechanisms. These disorders share features such as long-range underconnectivity and short-range overconnectivity in brain networks, reflecting similar patterns of neural dysfunction [8]. Long-range underconnectivity refers to weak connections between distant brain regions [9]. Conversely, short-range overconnectivity describes enhanced connections within local brain regions [10]. This pattern reflects distinct neural dysfunctions that can manifest across different conditions. Consequently, it has been hypothesised that ASD, ADHD, and TS may represent a spectrum of related neurodevelopmental disorders, collectively termed “abnormal connectivity spectrum disorders” [11].

Connective tissue diseases are a group of disorders impacting structures such as tendons, ligaments, cartilage, and the extracellular matrix. Several symptoms have a potential association with connective tissue disorders. Chronic fatigue and muscle pain are prevalent in autoimmune diseases and connective tissue disorders [12]. The excessive sweating of hands and feet can be associated with connective tissue disorders [13,14]. Gastrointestinal symptoms (constipation and diarrhoea) are often seen in connective tissue disorders [15,16]. Hypersensitivity to sensory stimuli, including tactile, visual, and auditory inputs, is linked to various connective tissue disorders, such as Ehlers–Danlos syndrome. Sensory processing differences are observed in approximately 90% of individuals with ASD, particularly in response to tactile stimuli. Recent research using ASD mouse models has shed light on the neurobiological mechanisms underlying these sensory alterations [17].

In addition, connective tissue plays a crucial role in supporting the structure and function of the nervous system [18]. The interaction between brain network connectivity disorders and connective tissue abnormalities suggests the existence of a complex relationship in which structural issues may influence functional outcomes [19]. A recent study utilising in vivo confocal microscopy revealed significant changes in the corneal connective tissue structure of adults with ASD compared to typically developing controls [20]. A broader perspective on the central and peripheral connectivity alterations characterising NDDs is proposed through the “Connectivome Theory” [21]. This theory underscores the role of connective tissue in various organs, highlighting its multifunctional properties, including structural support, connection, nourishment, regulation, and modulation among different cellular elements. Therefore, abnormalities in glial function or extracellular matrix composition and alterations in connective tissue can hinder proper neuronal wiring [22].

The primary objective of this study is to examine the prevalence of pathologies associated with connective tissue alterations in individuals diagnosed with ASD, ADHD, or TS and to compare these findings with those from a healthy control group. A secondary aim is to evaluate whether these symptoms are observed at a significant rate among the parents of affected individuals.

## 2. Materials and Methods

The questionnaire (see Appendix A), designed to investigate symptoms associated with connective tissue disorders, was administered to the families of 120 individuals diagnosed with NDDs, specifically ASD, ADHD, and TS, as well as to a control group attending the Child and Adolescent Neuropsychiatry Outpatient Clinics at the University Hospital of Verona, Italy.

The data collection period spanned from December 2019 to January 2022. Parents or caregivers actively participated in the study by responding to structured questionnaires, contributing to data acquisition.

This study was approved by the Ethical Committee of the University Hospital of Verona under the following codes: CESC 2243 (Paediatric Clinic, University Hospital of Verona) and CESC 2242 (Child and Adolescent Neuropsychiatry Outpatient Clinics, University Hospital of Verona). Written informed consent was obtained from each participant’s parents.

The questionnaire gathered sociodemographic data and assessed the presence of 18 symptom categories in both the patients and their parents. These categories were as follows:

- Varicose veins, vasculitis, and haemorrhoids;
- Striae rubrae, skin irregularities, or redness;
- Excessive sweating of the hands and feet;
- Joint inflammation and rheumatism;
- Joint dislocations and subluxations/dislocations;
- Joint pain involving 1 to 3 large joints lasting for more than 3 months;
- Back pain, transient muscle pain in the limbs, or chronic fatigue;
- Hip dysplasia, scoliosis, or kyphosis;
- Inguinal, umbilical, abdominal, or disc hernias;
- Flat feet;
- Constipation, diarrhoea, or alternating bowel patterns;
- Heartburn, gastroesophageal reflux, or hiatal hernia;
- Use of orthodontic appliances;
- Tactile, visual, auditory, olfactory, or gustatory hypersensitivity;
- Myopia or drooping eyelids, including unilateral or bilateral ptosis;
- Immune and/or autoimmune diseases.

The same questionnaire was also administered to a control group comprising 44 families with typically developing, healthy children attending the clinic.

### Statistical Analysis

Continuous Gaussian variables were summarised as mean values with standard deviations (S.D.). Categorical variables were described using counts and percentages.

The presence of symptoms was compared across controls, TS, ASD, and ADHD groups using logistic regression models. In these models, the dependent variable was the presence of the symptom, while case–control status served as the independent variable. Age and sex were included as covariates to adjust the estimates.

To address issues of separation and potential convergence difficulties caused by the low prevalence of certain symptoms, Firth’s penalised maximum likelihood method was employed in the logistic regression analyses [23,24].

Statistical analyses were conducted using the STATA 16.0 software package (www.stata.com). A significance threshold of 5% was applied for all statistical tests.

## 3. Results

A total of 164 participants were included in the study. Of these, 44 (26.8%) were healthy controls, while the remaining 120 (73.2%) were cases: there were 48 (29.3%) with ASD, 36 (22.0%) with ADHD, and 36 (22.0%) with TS. The mean age of the cases was 10.1 years (S.D.: 3.6), compared to the 9.5 years (S.D.: 2.5) observed for the controls.

The control group was evenly distributed by sex, with 22 males (50.0%) and 22 females (50.0%). In contrast, most cases were male, comprising 110 participants (91.7%).

**Figure 1:**
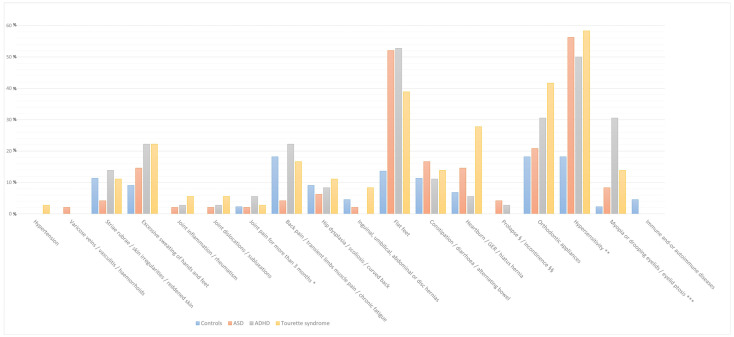
The bar graph illustrates the percentage prevalence of symptoms among children in the control group and those with neurodevelopmental disorders, including autism spectrum disorder, attention deficit hyperactivity disorder, and Tourette syndrome. The categories are colour-coded as follows: ASD (orange), ADHD (green), Tourette syndrome (yellow), and controls (blue). Abbreviations: * Joint pain involving 1 to 3 large joints lasting for more than 3 months; **, Tactile/visual/auditory/olfactory or gustatory hypersensibility; ***, Myopia or drooping eyelids/unilateral or bilateral eyelid ptosis.

The analysis presented in Table 1 demonstrates that flat feet are significantly more prevalent among individuals with ASD (52.1%), ADHD (52.8%), and TS (38.9%) compared to the control group (13.6%), indicating a strong association with the NDDs under investigation. Heartburn, gastro-oesophageal reflux, and hiatus hernia are notably more frequent in individuals with TS (27.8%) relative to the other groups. Similarly, the use of orthodontic appliances is more common in subjects with TS (41.7%) compared to the other cohorts. Hypersensitivity is markedly more prevalent in individuals with ASD (56.3%), ADHD (50.0%), and TS (58.3%) compared to controls (18.2%), further supporting the existence of a strong correlation with the NDDs included in the study. Additionally, myopia and ptosis are observed more frequently in individuals with ADHD (30.6%) compared to the other groups (Table 1, Figure 1).

The analysis in Table 2 indicates that individuals with ASD exhibit a lower tendency to experience back pain, transient muscle pain in the limbs, and chronic fatigue (OR 0.25; 95% CI: 0.053–1.16; *p* = 0.076). The data also reveal a strong association between flat feet and various NDDs, including ADHD (OR 6.73; 95% CI: 2.097–21.63; *p* = 0.001), ASD (OR 7.20; 95% CI: 2.438–21.23; *p* < 0.001), and TS (OR 3.70; 95% CI: 1.107–12.34; *p* = 0.034). Additionally, there is a potential trend towards the increased use of orthodontic appliances in TS (OR 3.20; 95% CI: 0.887–11.51; *p* = 0.076).

A significant association between hypersensitivity and NDDs was also identified. Individuals with hypersensitivity demonstrate a higher propensity for ADHD (OR 4.11; 95% CI: 1.385–12.19; *p* = 0.011), ASD (OR 5.90; 95% CI: 2.160–16.12; *p* = 0.001), and TS (OR 5.35; 95% CI: 1.738–16.47; *p* = 0.003), confirming that there is a robust association between hypersensitivity and these disorders. Finally, the analysis revealed that there was a significant association between myopia or eyelid ptosis and ADHD (OR 13.12; 95% CI: 1.859–92.56; *p* = 0.01).

**Figure 2:**
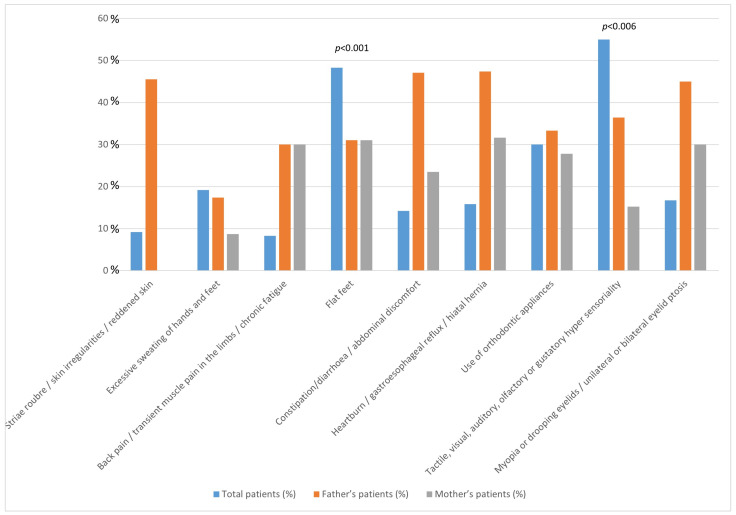
The figure presents the percentage distribution of symptoms among all patients, their fathers, and their mothers. The statistically positive significance of the observed associations is shown.

Given that both flat feet and hypersensitivity were more prevalent in each NDD under investigation, we performed a familial analysis across the entire cohort of parents (Table 3; Figure 2). Flat feet were common (OR 8.3) among fathers (31.0%), mothers (31.0%), and patients (43.8%; *p* < 0.001). The symptoms of tactile, visual, auditory, olfactory, or gustatory hypersensitivity were more frequent (OR 3.31) among fathers (36.4%) compared to mothers (15.2%) and were associated with a high prevalence of the symptoms in patients (55%; *p* = 0.006).

## 4. Discussion

In our study, we identified a strong association between flat feet, hypersensitivity, and the neuropsychiatric conditions of ASD, ADHD, and TS. The use of orthodontic appliances was more frequent in individuals with TS, and myopia and ptosis were notably prevalent in those with ADHD. Additionally, flat feet and hypersensitivity were more common among the parents affected by the NDDs under investigation.

Recent studies have indicated that there is a potential link between NDDs and connective tissue-related symptoms. The underlying mechanisms linking NDDs and connective tissue-related symptoms may involve immune dysregulation, chronic inflammation, and vascular issues that impact brain function [25,26]. For example, a condition characterised by cognitive and behavioural challenges and notable physical features linked to connective tissue disorders is Fragile X Syndrome. Individuals with Fragile X Syndrome often exhibit distinct physical traits associated with connective tissue dysregulation, such as joint hypermobility, hyperextensible skin, and increased tissue fragility [27,28].

Our findings revealed a significantly higher prevalence of flat feet in individuals with ASD (52.1%), ADHD (52.8%), and TS (38.9%) compared to the control group (13.6%). In a study analysing children aged 3 to 10 years, the overall prevalence of flat feet was reported to be 15.7% [29]. A systematic review indicated that the detection rate of flat feet in children over the past two decades was approximately 25% [30]. Previous research indicated that 52–53% of children with ADHD had exhibit mild to severe flat feet, in contrast to only 8–13% of their typically developing peers [31].

The causes of flat feet can be categorised as either congenital or acquired, but identifying the specific aetiology in paediatric cases is challenging. Flat feet may present as an isolated condition or as part of a broader syndrome [32]. For instance, certain congenital disorders, including Ehlers–Danlos syndrome, are characterised by both flat feet and NDDs due to underlying connective tissue abnormalities [33]. Notably, genetic syndromes impacting connective tissue, such as Marfan syndrome and Ehlers–Danlos syndrome, are linked to neurodevelopmental challenges and mitral cardiovascular issues, including valve prolapse and aortic aneurysms [34,35,36]. The shared pathophysiological mechanism underlines the clinical overlap between connective tissue anomalies and associated cardiovascular complications.

In connective tissues, elastic fibres play a crucial role in the extracellular matrix, contributing to the elasticity and resilience of tissues. These fibres give connective tissues elasticity and resilience [37]. Although no direct evidence links flat feet to alterations in the extracellular matrix, the structural integrity of the foot is mainly dependent on connective tissues, primarily composed of extracellular matrix components.

In our study, we observed that hypersensitivity was significantly more prevalent in individuals with TS (58.3%; OR 5.35, 95% CI 1.738–16.47), ASD (56.3%; OR 5.9, 95% CI 2.160–16.12), and ADHD (50.0%; OR 4.11, 95% CI 1.385–12.19) compared to controls (18.2%). In a representative sample of elementary school-aged children (ages 7–11), 16% of parents reported that their children had at least four tactile or auditory sensations [38]. Among children with ASD, the prevalence of sensory abnormalities was significantly higher at 53.6% compared to 8.0% in non-ASD children [39].

In the context of ASD, hypersensitivity refers to an increased sensitivity to stimuli such as sound, light, touch, taste, and smell. Research has suggested that this heightened sensitivity is associated with abnormalities in glial cells, which may disrupt the development of the myelin sheath, leading to delays in response times [40,41]. There is a substantial body of literature supporting the association between sensory alterations and ASD [21,42,43,44]. Tactile hypersensitivity and auditory hypersensitivity have been identified as predictors of an ASD diagnosis [39]. Moreover, connective tissue plays a crucial role in the structure and function of the outer, middle, and inner ear and the central auditory pathways.

In our study, myopia and ptosis were more prevalent in subjects with ADHD (30.6%; OR 13.12, 95% CI 1.859–92.56) compared to those with TS (13.9%) and ASD (8.3%) and compared to controls (2.3%). A systematic review covering data from 2000 to 2022 reported the overall pooled prevalence of childhood myopia was 5.2% [45].

Previous studies have not identified a significant association between myopia and ptosis [46]. However, individuals with ASD often exhibit defects in oculomotor activity and the pupillary sphincter response to the light reflex [47] and frequently display refractive deficits [48]. The most common ocular issues in ASD include difficulties with eye alignment (convergence insufficiency) and refractive errors [44]. The connective tissue in the eyes plays a crucial role in supporting the blood vessels and nerves that comprise the retina [49]. Additionally, the corneal–scleral framework primarily comprises connective tissue [49,50].

The use of orthodontic appliances was more common in individuals with TS (41.7%) compared to other groups and controls (OR 3.20, 95% CI 0.887–11.51). A study conducted in Germany revealed that approximately 33.5% of children aged 11–14 years were undergoing orthodontic treatment [51]. In Denmark, a retrospective study indicated that 27% of children were undergoing orthodontic appliance therapy [52]. Factors influencing treatment frequency included age, gender, and socio-economic status.

Orthodontic appliances (oral orthotics, occlusal splints) have been investigated as potential treatments for reducing tics in individuals with TS. Some studies have explored the use of customised oral splints and dental orthodontic devices to manage tics [53,54]. In recent years, reports have suggested that dental orthodontic devices, typically used for treating temporomandibular joint (TMJ) disorders, may also effectively reduce tics when worn by individuals with TS [54].

Family history is strongly associated with the risk of ASD. The individual risk of ASD increases with closer genetic relationships [3]. A reanalysis of a previous study on the familial risk of ASD estimated that 83% of the risk could be attributed to genetic factors, suggesting that genetics play a pivotal role in the development of ASD [55]. In the familial analysis of our study, which included patients diagnosed with ASD, ADHD, and TS, flat feet were observed frequently in fathers (31%), mothers (31%), and patients (48.3%). Sensory hypersensitivity, which was commonly observed in patients (55%), was more prevalent in fathers (24%) compared to mothers (10%). Myopia and ptosis, which were present in 16.7% of the patients, were more frequently found in fathers (45%) than in mothers (30%). To date, the search results have not provided specific data regarding the prevalence of hypersensitivity and flat feet in the parents of children with NDDs.

The recent literature suggests that ptosis, myopia, and flat feet can manifest as features of connective tissue disorders. Flat feet have been found to be significantly more prevalent in individuals with ASD, while myopia and ptosis are more common among children with ADHD. Additionally, the use of orthodontic appliances has been observed to be more frequently in individuals with TS, which may be linked to underlying connective tissue abnormalities. Notably, orthodontic appliance use has been associated with connective tissue disorders, particularly in conditions like Ehlers–Danlos syndrome [56]. These findings indicate that ptosis, flat feet, and the use of orthodontic appliances, as potential manifestations of connective tissue disorders, may have significant genetic and phenotypic overlaps with specific NDDs.

In the context of ASD, ADHD, and TS, abnormalities in connective tissue may represent a shared underlying factor. This hypothesis is supported by emerging research that underscores the interconnectedness of these NDDs through physical manifestations [57,58].

Official symmetry refers to the balanced arrangement of features, which is significant in understanding neurodevelopmental disorders (NDDs) such as ASD, ADHD, and TS [59]. Research highlights the role of brain symmetry in diagnosing and treating these disorders [59], linking structural abnormalities to symptom severity [60]. Associations between NDDs and physical traits, like flat feet and hypersensitivity, underscore the need to explore genetic and developmental pathways to achieve better outcomes [61,62]. This holistic perspective integrates behavioural and physical characteristics as interconnected aspects of NDDs.

Several limitations may affect the validity and generalisability of the present study. The small sample size of each NDD subgroup and the gender imbalance may introduce bias into the results. Clinic-based recruitment could lead to selection bias, and the control group may not represent the general population. Additionally, reliance on self-reported questionnaires without evaluating their reliability and validity may result in recall bias. The subjective nature of some symptoms also lacks clinical verification. The use of multiple testing increases the risk of false positives. This study does not account for all potential confounders, and its cross-sectional design prevents the establishment of causal inferences. Furthermore, while the familial analysis suggests a genetic component, it is limited by the absence of genetic testing.

Finally, our study did not directly compare the prevalence of physical findings, such as flat feet, between individuals with NDDs and the general population. This limits the generalizability of our findings. Future research should focus on direct comparative analyses to clarify these associations.

## 5. Conclusions

In conclusion, the study identified significant associations between certain NDDs (ASD, ADHD, and TS) and physical symptoms, such as flat feet and sensory hypersensitivity. These symptoms were also frequently observed in parents, suggesting the existence of a strong familial component. This finding implies that overlapping aetiological factors may manifest in various ways, with connective tissue abnormalities potentially serving as a common underlying factor. Further research is required to confirm and expand upon these findings.

## Figures and Tables

**Table 1:** The table shows the prevalence of symptoms in four groups: ASD (autism spectrum disorder), ADHD (attention deficit hyperactivity disorder), TS (Tourette syndrome), and controls (individuals without specific diagnoses). The data are expressed as the percentage of affected individuals in each group and the percentage variation (Δ%) compared to the controls.

Symptoms	ASD (%)	Δ%	ADHD (%)	Δ%	TS (%)	Δ%	Controls (%)
Hypertension	0.0	0	0.0	0	2.8	2.8	0.0
Varicose veins/vasculitis/haemorrhoids	2.1	2.1	0.0	0	0.0	0	0.0
Striae rubrae/skin irregularities/reddened skin	4.2	−7.2	13.9	2.5	11.1	−0.3	11.4
Excessive sweating of hands and feet	14.6	5.5	22.2	13.1	22.2	13.1	9.1
Joint inflammation/rheumatism	2.1	2.1	2.8	2.8	5.6	5.6	0.0
Joint dislocations/subluxations/dislocations	2.1	2.1	2.8	2.8	5.6	5.6	0.0
Joint pain involving 1 to 3 large joints lasting for more than 3 months	2.1	−0.2	5.6	3.3	2.8	0.5	2.3
Back pain/transient muscle pain in the limbs/chronic fatigue	4.2	−14	22.2	4	16.7	−1.5	18.2
Hip dysplasia/scoliosis/curved back	6.3	−2.8	8.3	−0.8	11.1	2	9.1
Inguinal/umbilical, abdominal, or disc hernias	2.1	−2.4	0.0	−4.5	8.3	3.8	4.5
Flat feet	52.1	38.5	52.8	39.2	38.9	25.3	13.6
Constipation/diarrhoea/alternating bowel	16.7	5.3	11.1	−0.3	13.9	2.5	11.4
Heartburn/gastroesophageal reflux/hiatal hernia	14.6	7.8	5.6	−1.2	27.8	21	6.8
Rectal or uterine prolapse/urinary and/or faecal incontinence	4.2	4.2	2.8	2.8	0.0	0	0.0
Use of orthodontic appliances	20.8	2.6	30.6	12.4	41.7	23.5	18.2
Tactile/visual/auditory/olfactory or gustatory hypersensibility	56.3	38.1	50.0	31.8	58.3	34.8	18.2
Myopia or drooping eyelids/unilateral or bilateral eyelid ptosis	8.3	6	30.6	28.3	13.9	11.6	2.3
Immune and/or autoimmune diseases	0.0	−4.5	0.0	0	0.0	0	4.5

Legend: Δ%, percentage variation in each symptom compared to the controls.

**Table 2:** The table presents the results of the regression analysis used to assess the association between symptoms and autism spectrum disorder, attention deficit hyperactivity disorder, and Tourette syndrome.

Symptoms	ASD (OR, 95% CI)	*p* Value (ASD)	ADHD (OR, 95% CI)	*p* Value (ADHD)	TS (OR, 95% CI)	*p* Value (TS)
Hypertension	2.19 (0.021–228.08)	0.740	1.86 (0.011–306.46)	0.812	4.90 (0.033–734.81)	0.534
Varicose veins/vasculitis/haemorrhoids	3.58 (0.069–185.13)	0.526	1.26 (0.011–143.97)	0.924	1.09 (0.009–135.69)	0.973
Striae rubrae/skin irregularities/reddened skin	0.34 (0.062–1.91)	0.222	0.81 (0.188–3.45)	0.771	0.52 (0.107–2.54)	0.419
Excessive sweating of hands and feet	1.78 (0.451–7.01)	0.411	2.12 (0.523–8.56)	0.293	1.79 (0.422–7.62)	0.429
Joint inflammation/rheumatism	2.61 (0.065–105.07)	0.610	2.43 (0.059–99.60)	0.639	3.40 (0.082–141.57)	0.520
Joint dislocations/subluxations	2.79 (0.075–104.34)	0.578	3.10 (0.071–134.14)	0.557	4.82 (0.116–201.07)	0.409
Joint pain involving 1 to 3 large joints for more than 3 months	1.14 (0.083–15.65)	0.922	1.79 (0.151–21.20)	0.645	0.90 (0.053–15.23)	0.942
Back pain/transient muscle pain in the limbs/chronic fatigue	0.25 (0.053–1.16)	0.076	1.21 (0.353–4.12)	0.765	0.82 (0.211–3.15)	0.769
Hip dysplasia/scoliosis/curved back	0.91 (0.170–4.92)	0.915	0.84 (0.151–4.69)	0.846	0.99 (0.176–5.55)	0.988
Inguinal, umbilical, abdominal or disc hernias	0.55 (0.060–5.03)	0.596	0.18 (0.007–4.70)	0.306	1.21 (0.150–9.71)	0.860
Flat feet	7.20 (2.438–21.23)	<0.001	6.73 (2.097–21.63)	0.001	3.70 (1.107–12.34)	0.034
Constipation/diarrhoea/alternating bowel	2.07 (0.579–7.44)	0.263	1.45 (0.325–6.49)	0.625	1.85 (0.414–8.28)	0.421
Heartburn/gastroesophageal reflux/hiatus hernia	1.92 (0.460–7.98)	0.372	0.63 (0.105–3.75)	0.609	3.05 (0.697–13.37)	0.138
Rectal or uterine prolapse/urinary and/or faecal incontinence	4.34 (0.144–131.51)	0.399	2.58 (0.068–97.84)	0.609	0.71 (0.009–56.36)	0.880
Use of orthodontic appliances	2.02 (0.602–6.77)	0.255	2.36 (0.650–8.57)	0.192	3.20 (0.887–11.51)	0.076
Tactile, visual, auditory, olfactory or gustatory hypersensitivity	5.90 (2.160–16.12)	0.001	4.11 (1.385–12.19)	0.011	5.35 (1.738–16.47)	0.003
Myopia or drooping eyelids/unilateral or bilateral eyelid ptosis	3.18 (0.433–23.37)	0.255	13.12 (1.859–92.56)	0.010	5.01 (0.615–40.81)	0.132
Immune and/or autoimmune diseases	0.38 (0.018–8.18)	0.537	0.30 (0.010–9.11)	0.488	0.38 (0.011–13.29)	0.594

Legend: CI, confidence interval; OR, odds ratio; TS, Tourette syndrome. Yellow colour: statistically significant.

**Table 3:** The table presents a comparative analysis of the percentage distribution of symptoms among all patients, their fathers, and their mothers, alongside the statistical significance of the observed associations.

Symptoms		Controls	Overall Patients (ASD + ADHD + TS)	Father’s Patients	Mother’s Patients		Statistical Analysis	
		*n* (%)	*n* (%)	*n* (%)	*n* (%)	OR	*p*-Value	*p*-Overall
Totals		44	120					
Striae rubrae/skin irregularities/reddened skin	absent	39 (88.6)	109 (90.8)	15 (13.8)	7 (6.4)	2.44	0.206	0.330
	present	55 (11.4)	11 (9.2)	5 (45.5)	0 (0)			
Excessive sweating of hands and feet	absent	40 (90.9)	97 (80.8)	11 (11.3)	10 (10.3)	1.49	0.480	0.299
	present	4 (9.1)	23 (19.2)	4 (17.4)	2 (8.7)			
Back pain/transient muscle pain in the limbs/chronic fatigue	absent	36 (81.8)	110 (91.7)	21 (19.1)	19 (17.3)	2.08	0.283	0.862
	present	8 (18.2)	10 (8.3)	3 (30.0)	3 (30.0)			
Flat feet	absent	38 (86.4)	62 (51.7)	5 (8.1)	4 (4.5)	8.3	<0.001	<0.001
	present	6 (13.6)	58 (48.3)	18 (31.0)	18 (31.0)			
Constipation/diarrhoea/abdominal discomfort	absent	39 (88.6)	103 (85.8)	23 (22.3)	8 (7.8)	3.01	0.041	0.302
	present	55 (11.4)	17 (14.2)	8 (47.1)	4 (23.5)			
Heartburn/gastroesophageal reflux/hiatus hernia	absent	41 (93.2)	101 (84.2)	33 (32.7)	25 (24.8)	1.68	0.344	0.389
	present	3 (6.8)	19 (15.8)	9 (47.4)	6 (31.6)			
Use of orthodontic appliances	absent	36 (81.8)	84 (70.0)	23 (27.4)	10 (11.9)	1.48	0.372	0.109
	present	8 (18.2)	36 (30.0)	12 (33.3)	10 (27.8)			
Tactile, visual, auditory, olfactory or gustatory hypersensitivity	absent	36 (81.8)	54 (45.0)	8 (14.8)	3 (5.6)	3.31	0.006	<0.001
	present	8 (18.2)	66 (55.0)	24 (36.4)	10 (15.2)			
Myopia or drooping eyelids/unilateral or bilateral eyelid ptosis	absent	43 (97.7)	100 (83.3)	28 (28.0)	26 (26.0)	1.27	0.635	0.059
	present	1 (2.3)	20 (16.7)	9 (45.0)	6 (30.0)			

Legend: ASD, autism spectrum disorder; ADHD, attention deficit hyperactivity disorder; NDDs, neurodevelopmental disorders; OR, odds ratio; TS, Tourette’s syndrome. Yellow colour: statistically significant; green colour: borderline significance (0.05 < *p* < 0.100).

## Data Availability

Data are unavailable due to privacy and ethical restrictions.

## Appendix A

Questionnaire used for the study, in English language and Italian language.

Paediatric Laxity Questionnaire

Subject’s gender:
Male
Female
Subject’s diagnosis:
Autism
ADHD
Tourette’s syndrome
Control
How many brothers does the subject have?
How many sisters does the subject have?
Has the child or their parents had high blood pressure on two or three occasions?
Has the child or their parents had varicose veins, vasculitis, or haemorrhoids?
Has the child or their parents had stretch marks, skin discolouration, or unexplained reddened skin?
Has the child or their parents had excessive sweating of the palms and/or soles of the feet?
Has the child or their parents had joint inflammation or rheumatism (bursitis, tenosynovitis, etc.)?
Has the child or their parents had dislocations, subluxations, or luxation of one or more joints on more than one occasion?
Has the child or their parents had joint pain involving 1 to 3 large joints for more than 3 months?
Has the child or their parents had back pain, transient muscle aches in the limbs (e.g., growing pains), or symptoms of chronic fatigue?
Has the child or their parents had hip dysplasia, scoliosis, or a curved spine?
Has the child or their parents had inguinal, umbilical, or abdominal hernias, or herniated discs?
Has the child or their parents had flat feet?
Has the child or their parents had constipation, diarrhoea, or alternating bowel habits?
Has the child or their parents had heartburn, gastro-oesophageal reflux, or a hiatus hernia?
Has the child or their parents had rectal or uterine prolapse, or urinary or faecal incontinence?
Has the child or their parents worn or wear orthodontic appliances?
Has the child or their parents had hypersensitivity to touch (feeling tags on trousers, discomfort with a belt, feeling of tight trousers), sight (perception of bright lights, glare), hearing (perception of reverberations, amplified noises), smell (heightened perception of odours, dysosmia), or taste?
Has the child or their parents had a diagnosis of myopia or drooping eyelids (ptosis), either unilateral or bilateral? Has the child or their parents had any immune or autoimmune diseases? (e.g., type 1 diabetes, coeliac disease, thyroiditis, rheumatoid arthritis, Crohn’s disease, polyneuropathy, multiple sclerosis, etc.)
Has the child or have the parents had, or do they currently have, any immune or autoimmune diseases? (e.g., type 1 diabetes, coeliac disease, thyroiditis, rheumatoid arthritis, Crohn’s disease, polyneuropathies, multiple sclerosis, etc.)

Questionario lassità pediatrica (Italian version)

Genere del soggetto:
Maschio
Femmina
Patologia del soggetto:
Autismo
ADHD
Sindrome di Tourette
Controllo
Quanti fratelli ha il soggetto?
Quante sorelle ha il soggetto?
Il bambino o i genitori hanno presentato o presentano ipertensione arteriosa in due o tre rilevazioni?
Il bambino o i genitori hanno presentato o presentano vene varicose, vasculiti o emorroidi?
Il bambino o i genitori hanno presentato o presentano striae rubre, smagliature e segni cutanei, cute arrossata senza apparente causa?
Il bambino o i genitori hanno presentato o presentano un eccesso di sudorazione ai palmi delle mani e/o alle piante dei piedi?
Il bambino o i genitori hanno presentato o presentano infiammazioni o reumatismi articolari (borsiti, tenosinoviti, ecc.)?
Il bambino o i genitori hanno presentato o presentano dislocazioni, sublussazioni o lussazioni di una o più articolazioni in più di una occasione?
Il bambino o i genitori hanno presentato o presentano dolore articolare coinvolgente da 1 a 3 grosse articolazioni lamentato per più di 3 mesi?
Il bambino o i genitori hanno presentato o presentano dolore alla schiena, dolori muscolari transitori agli arti (ad es. dolori della crescita) o sintomi da affaticamento cronico?
Il bambino o i genitori hanno presentato o presentano displasia dell’anca, scoliosi o dorso curvo?
Il bambino o i genitori hanno presentato o presentano ernie inguinali, ombelicali, addominali oppure ernie discali?
Il bambino o i genitori hanno presentato o presentano piedi piatti?
Il bambino o i genitori hanno presentato o presentano stipsi, diarrea o alvo alterno?
Il bambino o i genitori hanno presentato o presentano pirosi retrosternale, reflusso gastroesofageo o ernia iatale?
Il bambino o i genitori hanno presentato o presentano prolassi rettali/uterini oppure incontinenza urinaria e/o fecale?
Il bambino o i genitori hanno utilizzato o utilizzano apparecchi ortodontici?
Il bambino o i genitori hanno presentato o presentano ipersensorialità a livello tattile (percepiscono etichette dei pantaloni, fastidio per cintura, senso di pantaloni stretti), a livello visivo (percezione di luci intense, abbagliamenti), a livello uditivo (percezione di rimbombi, rumori accentuati), a livello olfattivo (percezioni accentuate di odori, disosmie) a livello gustative?
Il bambino o i genitori hanno presentato o presentano un quadro oculistico di miopia oppure un rilievo di palpebre cadenti o ptosi palpebrale mono o bi laterale?
Il bambino o i genitori hanno presentato o presentano malattie immunitarie e/o autoimmunitarie? (es. diabete di tipo 1, celiachia, tiroiditi, artrite reumatoide, morbo di Crohn, polineuropatie, sclerosi multipla, etc.)
